# Harlequin Ichthyosis in a Preterm Neonate: A Case Report

**DOI:** 10.7759/cureus.97912

**Published:** 2025-11-27

**Authors:** Alexandra Maria Ulmeanu, Diana Maria Damian, Roxana Maria Nemes, Cristina Aurelia Ionita

**Affiliations:** 1 Neonatology, Regina Maria Hospital, Bucharest, ROU; 2 Pulmonology Department, University Titu Maiorescu, Bucharest, ROU

**Keywords:** genetic disorder, harlequin ichthyosis, neonate, prematurity, surfactant deficiency

## Abstract

Harlequin ichthyosis is a rare autosomal recessive disorder characterized by severe hyperkeratosis and impaired skin barrier function, often associated with high neonatal mortality. We present the case of a preterm neonate born by cesarean section at 32 weeks of gestation with classical features of Harlequin ichthyosis, confirmed by genetic testing. Despite intensive neonatal and dermatologic management, the patient developed multiorgan complications and died on the 30th day of life. This report emphasizes the importance of early diagnosis, supportive care, and genetic counseling for affected families.

## Introduction

Harlequin ichthyosis (HI) is the most severe form of a larger group of disorders known as autosomal recessive congenital ichthyosis (ARCI). This group includes other, typically milder, phenotypes such as lamellar ichthyosis (LI), congenital ichthyosiform erythroderma (CIE), and pleomorphic ichthyosis (PI). The primary features include hyperkeratosis, dryness, flaking, and peeling of the skin, with increased transepidermal water loss due to abnormalities in the stratum corneum structure. The incidence is approximately one in 300,000 births, with high mortality primarily due to infection or respiratory failure [1,2]. HI is caused by mutations in the ABCA12 gene, which is responsible for lipid transport essential for skin barrier function [3,4]. Recent studies have identified multiple novel ABCA12 mutations and described genotype-phenotype correlations within the spectrum of autosomal recessive congenital ichthyosis, including Harlequin ichthyosis [3-5]. These data come from published cohorts and case series and are cited here as background, not as specific findings in our patient. Early diagnosis is critical for optimal management and genetic counseling [6].

## Case presentation

**Figure 1:**
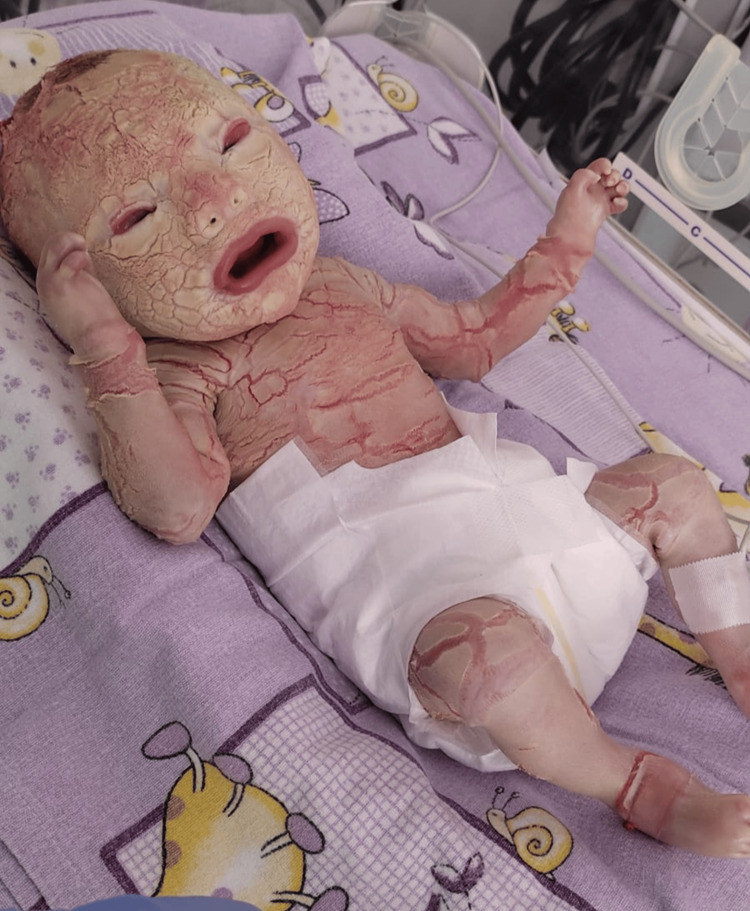
Day 1 image

A 35-year-old Caucasian woman, gravida II, para I, at 32 weeks and four days of gestation, presented in the emergency room with labor pain and ruptured membranes. Her obstetric history included one spontaneous abortion. The parents were a non-consanguineous couple with different blood types (mother O positive, father B positive). Pregnancy monitoring was unremarkable, with no infections or abnormal fetal morphology detected. Corticosteroids were administered for fetal lung maturation. After 35 hours, a female neonate weighing 2000 g was delivered by cesarean section with Apgar scores of 7 and 8 at one and five minutes (Figure 1).

At birth, the neonate presented with armor-like thickened yellowish scales with deep erythematous fissures, ectropion, eclabium, flattened nasal bridge, rudimentary ears, and absence of eyebrows, eyelashes, and hair, as we can see in Figure 1. The diagnosis of Harlequin ichthyosis was made clinically [7]. The baby was transferred to the neonatal intensive care unit (NICU) and placed in a humidified incubator (70-80% humidity) with continuous cardiorespiratory monitoring, including heart rate, respiratory rate, and pulse oximetry. The infant initially did not require mechanical ventilation or continuous positive airway pressure (CPAP) and was managed with supplemental oxygen delivered via the incubator to maintain adequate oxygen saturation. An umbilical venous catheter was inserted shortly after admission, and parenteral nutrition was initiated within the first hour of life, as the baby was unable to suck and nasogastric tube insertion was not initially feasible until day two of life. Empiric intravenous broad-spectrum antibiotics were started according to local NICU protocol, along with intravenous paracetamol for analgesia and fever control. During the second week of life, the infant developed fever and clinical deterioration, with elevated C-reactive protein and procalcitonin levels, highly suggestive of systemic infection. Antibiotic therapy was escalated, and antifungal treatment was added.

**Figure 2:**
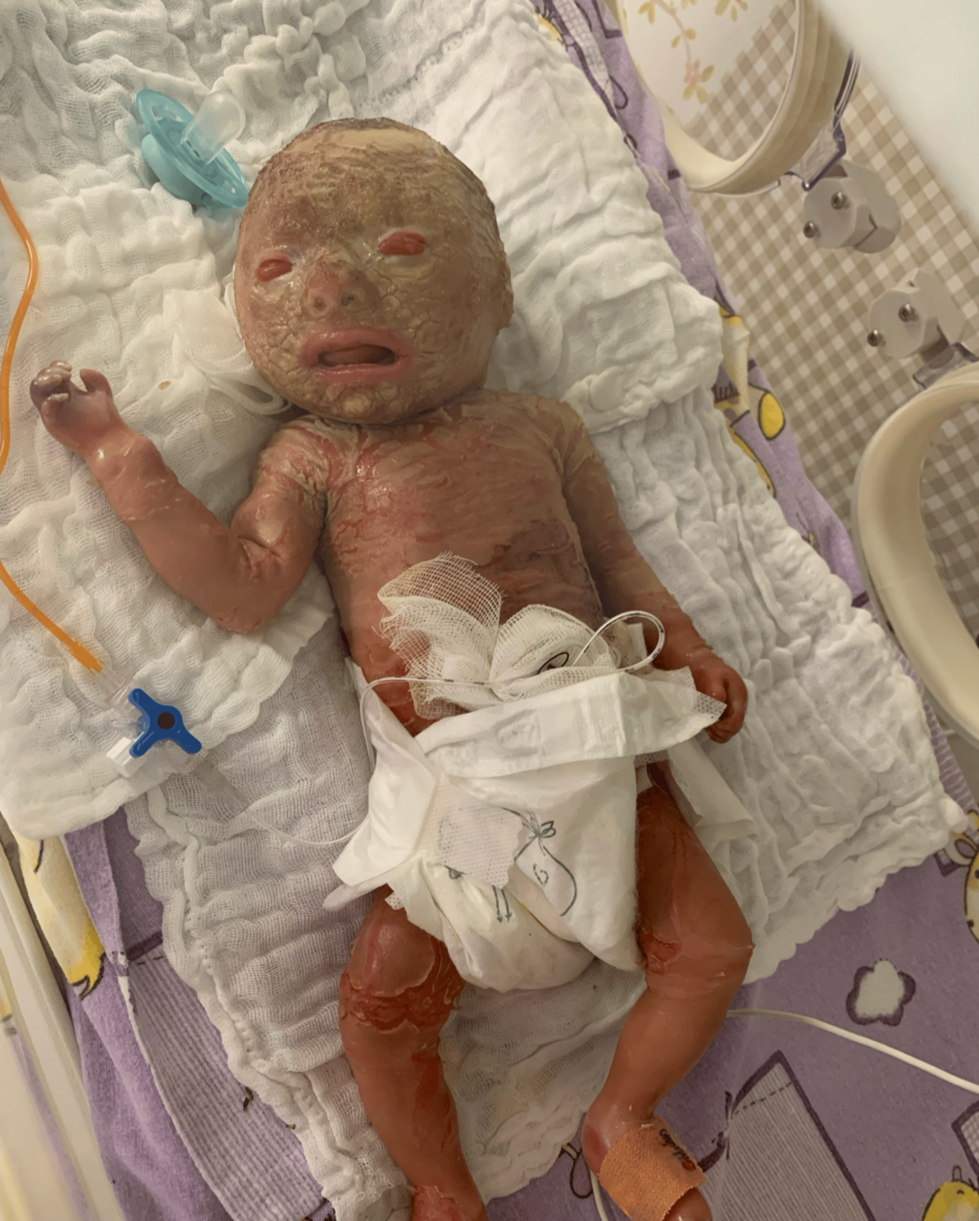
Day 10 image

Systemic retinoids were not initiated, following the recommendation of the consulting dermatologist to await genetic confirmation of the ABCA12 mutation and considering the infant’s severe general condition and ongoing infection (Figure 2). After initiation of intensive skin care with frequent application of lubricants and emollients, by around day 10 of life, the hyperkeratotic plates had become more softened and hydrated, adopting a yellowish-brown color and beginning to detach gradually. The underlying skin appeared bright red, more hydrated, but still scaly and fragile, with persistent fissures and areas at risk of infection. Despite the improvement in skin hydration, the overall clinical condition remained severe.

Genetic testing for ABCA12 was performed on day 9 of life, with written informed consent from the parents, and identified a homozygous pathogenic variant c.179G>C, p.(Arg60Pro) in the ABCA12 gene, confirming the diagnosis of Harlequin ichthyosis. Laboratory tests revealed early hypoglycemia, metabolic imbalances, and neonatal anemia requiring transfusion. Dermatologic management included daily saline cleansing, emollient application, topical antibiotics, and ocular protection. Retinoids were not used. Despite intensive supportive care, the skin remained extremely rigid with areas of necrosis and bleeding fissures. Around day 30 of life, the neonate developed recurrent episodes of oxygen desaturation and bradycardia. Oxygen concentration in the incubator was maximized; however, respiratory effort remained poor. Endotracheal intubation was attempted, but due to marked facial and cervical skin rigidity and limited mouth opening, intubation was not technically possible. The infant died shortly thereafter despite resuscitation efforts.

## Discussion

Harlequin ichthyosis is one of the most severe forms of autosomal recessive congenital ichthyosis (ARCI). It is caused by mutations in the ABCA12 gene, which encodes a lipid transporter necessary for the formation of the lipid barrier in the stratum corneum [3]. Defects in ABCA12 disrupt lamellar granule formation, leading to impaired lipid transport and a compromised epidermal barrier [8]. The phenotype is often lethal due to complications from infection, dehydration, and respiratory failure [2,5]. Recent advances in neonatal care, including humidity-controlled isolates, early antibiotic therapy, and oral retinoids, have improved survival [6,9]. Prenatal diagnosis of Harlequin ichthyosis is possible, particularly in high-risk pregnancies with a known family history of autosomal recessive congenital ichthyosis. Ultrasound findings in the late second or third trimester may include everted lips with a fixed open mouth, ectropion, limb contractures, and polyhydramnios. However, the true incidence of antenatal diagnosis based on ultrasound alone is low and not well defined, as most reports are limited to case reports and small series. In practice, definitive prenatal diagnosis relies on genetic testing for known ABCA12 mutations in families with an identified variant [4,10].

## Conclusions

Harlequin ichthyosis is a rare, life-threatening genetic disorder with poor prognosis. Management is multidisciplinary, requiring neonatology, dermatology, and genetics input. Early diagnosis, rigorous supportive care, and genetic counseling are essential to improve survival and guide family planning.
